# Insulin, dibutyryl-cAMP, and glucose modulate expression of patatin-like domain containing protein 7 in cultured human myotubes

**DOI:** 10.3389/fendo.2023.1139303

**Published:** 2023-03-22

**Authors:** Katarina Miš, Ana-Marija Lulić, Tomaž Marš, Sergej Pirkmajer, Maja Katalinić

**Affiliations:** ^1^ Institute of Pathophysiology, Faculty of Medicine, University of Ljubljana, Ljubljana, Slovenia; ^2^ Biochemistry and Organic Analytical Chemistry Unit, Institute for Medical Research and Occupational Health, Zagreb, Croatia

**Keywords:** PNPLA7, NRE, insulin, dexamethasone, forskolin, dibutyryl-cAMP, glucose, cultured human myotubes

## Abstract

Expression of patatin-like phospholipase domain containing protein 7 (PNPLA7), also known as neuropathy target esterase-related esterase (NRE), a lysophospholipase, increases with fasting and decreases with feeding in mouse skeletal muscle, indicating it is regulated by insulin, counterregulatory hormones, such as glucocorticoids and catecholamines, and/or nutrients. In cultured mouse adipocytes insulin reduces *Pnpla7* expression, underscoring the possibility that insulin regulates PNPLA7 in skeletal muscle. The first aim of this study was to establish whether PNPLA7 is functionally expressed in cultured human skeletal muscle cells. The second aim was to determine whether PNPLA7 is regulated by insulin, glucocorticoids, cAMP/protein kinase A pathway, and/or glucose. Cultured human skeletal muscle cells expressed *PNPLA7* mRNA and protein. Gene silencing of PNPLA7 in myoblasts reduced the phosphorylation of 70 kDa ribosomal protein S6 kinase and ribosomal protein S6 as well as the abundance of α1-subunit of Na^+^,K^+^-ATPase and acetyl-CoA carboxylase, indirectly suggesting that PNPLA7 is functionally important. In myotubes, insulin suppressed *PNPLA7* mRNA at 1 g/L glucose, but not at low (0.5 g/L) or high (4.5 g/L) concentrations. Treatment with synthetic glucocorticoid dexamethasone and activator of adenylyl cyclase forskolin had no effect on PNPLA7 regardless of glucose concentration, while dibutyryl-cAMP, a cell-permeable cAMP analogue, suppressed *PNPLA7* mRNA at 4.5 g/L glucose. The abundance of PNPLA7 protein correlated inversely with the glucose concentrations. Collectively, our results highlight that PNPLA7 in human myotubes is regulated by metabolic signals, implicating a role for PNPLA7 in skeletal muscle energy metabolism.

## 1 Introduction

The patatin-like phospholipase domain containing protein (PNPLA) family, characterized by the eponymous patatin domain, which was initially discovered in acyl hydrolase patatin, comprises nine enzymes (PNPLA1-9) with hydrolytic activity towards lipid substrates, such as phospholipids, triacylglycerols, as well as retinol- and cholesteryl-esters (1–5). PNPLAs participate in various biological processes, including regulation of energy metabolism, membrane homeostasis, and cell signalling (1–3, 6). Among PNPLAs, PNPLA6 (aka neuropathy target esterase (NTE), EC 3.1.1.5), localized to endoplasmic reticulum, and its homologue PNPLA7 (aka NTE-related esterase (NRE), EC 3.1.1.5), localized to endoplasmic reticulum and lipid droplets, constitute a phylogenetically distinct (NTE) subfamily (1, 2, 7–10). PNPLA6 is best known for its involvement in the organophosphate-induced delayed neuropathy, an axonopathy leading to degeneration of peripheral and central motor and sensory neurons (1, 7, 11, 12), although it is also important in neuronal development and brain function (7, 13–17). PNPLA7 is thought to participate in energy metabolism in peripheral tissues, such as liver and skeletal muscle (8–10, 18), but its regulation and putative metabolic function require further characterization.

PNPLA7, a lysophospholipase, preferentially hydrolyzes unsaturated species of lysophosphatidylcholine, an important signalling molecule and a building block of cellular membranes, thereby producing a glycerol-3-phosphocholine and a free fatty acid (8, 9, 19). In rodents, PNPLA7 is highly expressed in testis, heart and metabolic tissues, such as the liver, brown and white adipose tissue, and skeletal muscle (1, 8, 18–20). Deficiency of PNPLA7 in the liver reduces secretion of the very-low-density lipoproteins, potentially *via* destabilization of a direct PNPLA7 ligand apolipoprotein-E (21). Moreover, *Pnpla7* knock-out mice display hypoglycaemia, hypolipidaemia, as well as low consumption of energy, highlighting a role for PNPLA7 in whole-body energy metabolism (19). Expression of *PNPLA7* mRNA in testis, the adipose tissue and skeletal muscle in mice is reduced by feeding and increased by fasting (8, 19), while insulin suppressed *PNPLA7* mRNA levels in cultured 3T3-L1 adipocytes (8), consistent with the notion that PNPLA7 plays a role in the feed-fast cycle and metabolic homeostasis.

Skeletal muscles, which represent ~40% of body weight and ~20-30% of basal energy expenditure, are a major site of glucose and lipid metabolism and one of the most important insulin-responsive tissues (22, 23). Insulin resistance in skeletal muscle importantly contributes to dysregulation of glucose homeostasis in type 2 diabetes (24–26). While the role of PNPLA7 in skeletal muscles is not well understood, *Pnpla7*-/- mice display myopathic changes, including myofiber necrosis, degeneration and atrophy, indicating that PNPLA7 is important for skeletal muscle function (19, 27). However, despite its apparent functional relevance, whether insulin might regulate expression of PNPLA7 in skeletal muscle has not been determined. The effects of other metabolic hormones, such as glucocorticoids and catecholamines, which play a prominent role in regulation of energy metabolism, or fluctuations in extracellular glucose concentrations, all of which could plausibly contribute to modulation of PNPLA7 expression during feed-fast cycle, have also not been studied.

Importantly, most work concerning PNPLA7 has been performed using various animal models, which means that the insight into its role in humans is particularly deficient. The overarching objective of our study was therefore to provide new insights into the mechanisms by which PNPLA7 is regulated in human skeletal muscle. Our preliminary results suggested that cultured human myotubes might express PNPLA7 protein (28). The first specific aim of this study was therefore to establish whether PNPLA7 is functionally expressed in cultured human skeletal muscle cells. The second aim was to determine whether PNPLA7 is regulated by insulin, synthetic glucocorticoid dexamethasone, activators of cAMP/protein kinase A (PKA) signalling (forskolin, dibutyryl-cAMP), which were used to mimic catecholamine action *via* β-adrenergic receptors, and/or glucose.

## 2 Materials and methods

### 2.1 Materials

Cell culture flasks and plates were purchased from Sarstedt (Nümbrecht, Germany) or TPP (Transandigrn, Switzerland). Advanced MEM, DMEM, OptiMEM, GlutaMAX, MEM vitamin solution, fetal bovine serum (FBS), Fungizone (250 µg/mL of amphotericin B), gentamicin (10 mg/mL), trypsin-EDTA, Pierce Enhanced Chemiluminescence (ECL) Western Blotting substrate, High Capacity cDNA Reverse Transcription Kit, TaqMan Universal Master Mix with UNG and TaqMan gene expression assays were purchased from Thermo Fisher Scientific (Waltham, MA, U.S.). 4-12% Criterion Bis-Tris polyacrylamide gels, XT MES electrophoresis buffer, goat anti-mouse IgG-horseradish peroxidase conjugate, anti-rabbit IgG-horseradish peroxidase conjugate, PCR plates and PCR plate sealing films were purchased from Bio-Rad (Hercules, CA, U.S.). Amersham ECL Full-Range Rainbow Molecular Weight Marker was purchased from (Cytiva, U.S.). Polyvinylidene fluoride (PVDF) membrane, forskolin, dibutyryl-cAMP (db-cAMP), bovine serum albumin (BSA), and all other reagents were purchased from Merck/Sigma-Aldrich (Darmstadt, Germany). Insulin (Actrapid) was obtained from NovoNordisk (Denmark). E.Z.N.A HP Total RNA Kit was purchased from Omega Bio-Tek (Norcross, GA, U.S.). Dexamethasone was purchased from KRKA (Slovenia).

### 2.2 Ethical approvals

The primary human skeletal muscle cell cultures were prepared from the samples of *semitendinosus* muscle obtained during routine orthopaedic surgery as surgical waste, with informed written consent signed by the participants as described (29–32). Preparation of primary human skeletal muscle cells and experimental procedures involving these cells were approved by the Republic of Slovenia National Medical Ethics Committee (ethical approval no. 71/05/12 and 0120-698/2017/4).

### 2.3 Primary human skeletal muscle cell cultures and cell treatments

Human skeletal muscle cells were prepared from samples of the *semitendinosus* muscle as described (29–32). One experiment was performed using commercially available primary human skeletal myoblasts from Thermo Fischer Scientific (A12555). Primary myoblasts were expanded in growth medium (i.e. Advanced MEM supplemented with 10% FBS, 1% (v/v) GlutaMAX, 1% (v/v) MEM vitamin solution, 0.3% (v/v) Fungizone and 0.15% (v/v) gentamicin) at 37 ˚C in humidified air with 5% (v/v) CO_2_. Before the experiment, myoblasts were seeded into 12-well plates and cultured in the growth medium for 2 days. To induce differentiation, myoblasts were switched to differentiation medium (i.e. Advanced MEM supplemented with 2% (v/v) FBS, 1% (v/v) MEM vitamin solution, 0.3% (v/v) Fungizone and 0.15% (v/v) gentamicin). Experiments were performed on myotubes after 7-10 days of differentiation.

Experiments were performed in serum-free DMEM. To remove FBS and insulin, which is present in Advanced MEM, myotubes were washed with warm PBS (137 mM NaCl, 2.7 mM KCl, 10 mM Na_2_HPO_4_, 1.8 mM KH_2_PO_4_, pH=7.4) and switched to serum- and insulin-free DMEM (without antibiotics and antimycotics), which contained 0.5 g/L, 1 g/L, or 4.5 g/L glucose. Treatments with insulin (0.1 µg/mL and/or 10 µg/mL), dexamethasone (1 µM), forskolin (5 µM in DMSO), db-cAMP (200 µM) and/or vehicle lasted for 16 h. At the end of experiment myotube samples were lyzed and collected for the subsequent mRNA and protein analysis.

### 2.4 Neutralization of primary antibody against PNPLA7

Specificity of the primary antibody against PNPLA7 was determined by performing antibody neutralization experiment using Protein Epitope Signature Tag (PrEST) Antigen (3.6 mg/mL, Prestige Antibodies^®^ Atlas Antibodies, Merck, #APREST71921) with the following sequence: CEVGYQHGRTVFDIWGRSGVLEKMLRDQQGPSKKPASAVLTCPNASFTDLAEIVSRIEPAKPAMVDDESDYQTEYEEELLDVPRDAYADFQSTSAQQGSDLEDESSLRHRHPSLAFPKLSE. Briefly, primary antibody against PNPLA7 was incubated with the excess of PNPLA7 peptide antigen used for immunization. Incubation mixture contained 10 μL of PNPLA7 antibody (0.4 μg/mL), 1.1 μL of PNPLA7 PrEST antigen (0.8 μg/mL) and 29 μL of primary antibody buffer with 1 M urea. The mixture was incubated for 3.5 h at 22°C and constant shaking (300 rpm). After incubation, the mixture was diluted with the primary antibody buffer (containing 1 M urea) to the final volume of 5000 μL, the final antibody dilution thus reaching 1:500. The solution containing the neutralized PNPLA7 antibody solution was subsequently used for immunoblotting. To detect PNPLA7, homogenates of human *semitendinosus* muscle or human myotubes were used. Tissue homogenates, prepared in Laemmli buffer, were collected from our previous study (33).

### 2.5 Gene silencing of PNPLA7 in human myoblasts

The primary human skeletal muscle cells (myoblasts) were seeded into 6-well plates and cultured overnight in Advanced MEM supplemented with 10% (v/v) FBS, 1% (v/v) GlutaMAX and 1% (v/v) MEM vitamin solution, without antibiotics. The transfection was performed using Lipofectamine 2000 reagent (Thermo Fisher Scientific) and siRNA according to the manufacturer’s protocol. As a negative control, non-targeting scrambled RNA (siSCR) was used. Lipofectamine and siRNA were separately diluted in Opti-MEM and combined just before the addition to the cell culture medium. Final concentration of siRNA against PNPLA7 (Silencer™ Select Pre-Designed siRNA, #4392420, Ambion) and scrambled siRNA (ON-TARGETplus Non-Targeting Pool, D-001810-10-20, Dharmacon) in the medium was 10 nM. Final concentration of Lipofectamine was 2 ‰(v/v). After 24 h of incubation in the presence of the siRNA, the cell medium was replaced with the complete growth medium. Analyses of mRNA and protein levels were performed 48 and 72 h after the beginning of siRNA treatment, respectively.

### 2.6 Quantitative real-time PCR

At the end of experiment, cells were washed with sterile PBS solution and the total RNA was extracted with E.Z.N.A. HP Total RNA Kit. Concentration of isolated RNA was determined by micro-volume absorbance-based quantification (260 nm/280 nm) on Epoch™ spectrophotometer (BioTek Instruments, Inc., USA). RNA extracted from cell lysates was reverse transcribed to cDNA with High Capacity cDNA Reverse Transcription Kit and was performed in PTC-100 Programmable Thermal Controler (MJ Research Inc). qPCR was performed on Quant Studio 3 (Applied Biosystems, Thermo Fischer Scientific) using TaqMan^®^ Universal Master Mix II with UNG and TaqMan gene expression assays (PNPLA7 (Hs00295012_m1), IL-6 (Hs00174131_m1), acetyl-CoA carboxylase 1 (ACACA, Hs01046047_m1), acetyl-CoA carboxylase 2 (ACACB, Hs01565914_m1), β-actin (ACTB, Hs99999903_m1), 18S rRNA (Hs99999901_s1)) in a 96-well plate. β-actin (ACTB) and 18S rRNA were used as reference genes (endogenous controls). Results were calculated as gene expression ratios: (1+E*_reference_*)^Ct,^ *^reference^*/(1+E*_target gene_*)^Ct,^ *^target gene^* where E is the PCR efficiency and Ct is the threshold cycle. Since two reference genes were used for normalization, geometric mean of both expression ratios was calculated at the last step, while the efficiency of PCR was analyzed with the LinRegPCR software (34, 35), as described (31).

### 2.7 Immunoblotting

Immunoblotting was performed as described (29–32). Briefly, at the end of the experiment, cells were washed with ice-cold PBS three times, lysed in Laemmli buffer (62.5 mM Tris-HCl (pH=6.8), 2% (w/v) sodium dodecyl sulphate (SDS), 10% (w/v) glycerol, 5% (v/v) 2-mercaptoethanol and 0.002% (w/v) bromophenol blue) on ice and disrupted by sonification. Afterwards, samples were heated for 20 min at 56°C and 600 rpm and were frozen at -20°C until analysis. Proteins were resolved with SDS-PAGE on 4-12% polyacrylamide gels for up to 45 minutes at 200 V in XT-MES running buffer. Molecular weight marker Amersham Full Range Rainbow™ was used as a standard. Proteins were transferred to the PDVF membrane (0.45 μm) at 100 V for 60 minutes in transfer buffer (31 mM Tris, 0.24 M glycine, 10% (v/v) methanol and 0.01% (w/v) SDS) using wet electro transfer Criterion system (Bio-Rad, Hercules, CA, U.S.). After the transfer, the membranes were stained with Ponceau S (0.1% (w/v) in 5% (v/v) acetic acid) to evaluate sample loading and transfer. Actin was used as an additional loading control (see raw data in the **Supplement**). Then, the membranes were blocked with 7.5% (w/v) dry milk in the Tris-buffered saline with Tween-20 (TBST, 20 mM Tris, 150 mM NaCl, 0.02% (v/v) Tween-20, pH=7.5) for 1-2 hours at room temperature. For PNPLA7 detection, the membrane was blocked with 7.5% (w/v) dry milk in TBST with addition of 0.8% BSA. After blocking, membranes were washed three times in TBST and incubated with selected primary antibody (**Table 1**) in the primary antibody buffer (20 mM Tris, 150 mM NaCl, 0.1% (w/v) BSA, 0.1% (w/v) sodium azide, pH=7.5) overnight at 4°C. Membranes were then washed with TBST three times for 10 minutes and incubated with the horseradish peroxidase-conjugated secondary antibody with 5% (w/v) dry milk in the TBST for one hour at room temperature. After incubation in secondary antibody, membranes were washed in TBST three times for 10 minutes. Finally, membranes were incubated with ECL reagent for one minute and chemiluminescence of labelled proteins was visualized with the Fusion FX6 (Vilber, France) with the Evolution-Capt software. Bands were analyzed with Quantity One 1-D Analysis Software (Bio-Rad, Hercules, CA, U.S.).

**Table 1:** List of primary antibodies used for immunoblotting.

Antibody target	Catalogue No.	Source	Dilution	Manufacturer
**PNPLA7**	#HPA009130	Rabbit	1:400	Sigma Prestige, Merck KGaA, Germany
**p-AMPK Thr^172^**	#2535	Rabbit	1:1000	Cell Signaling Technology Inc, USA
**ACC**	#3676	Rabbit	1:1000	Cell Signaling Technology Inc, USA
**p-ACC Ser^79^**	#3661	Rabbit	1:1000	Cell Signaling Technology Inc, USA
**p-Akt Ser ^473^**	#4060	Rabbit	1:2000	Cell Signaling Technology Inc, USA
**p-PKA substrate**	#9621	Rabbit	1:1000	Cell Signaling Technology Inc, USA
**p-CREB Ser^133^**	#9198	Rabbit	1:1000	Cell Signaling Technology Inc, USA
**p-p70S6K Thr^389^**	#9205	Rabbit	1:1000	Cell Signaling Technology Inc, USA
**p-S6RP Ser ^235/236^**	#2211	Rabbit	1:1000	Cell Signaling Technology Inc, USA
**p-4E-BP1 Thr^37/46^**	#2855	Rabbit	1:1000	Cell Signaling Technology Inc, USA
**NKA α_1_**	#05-369	Mouse	1:2000	Upstate, Merck KGaA, Germany
**p-STAT3 Tyr^705^**	#9145	Rabbit	1:1000	Cell Signaling Technology Inc, USA
**Actin**	#A5441	Mouse	1:10000	Sigma Prestige, Merck KGaA, Germany

Secondary antibody: goat anti-rabbit IgG-horseradish peroxidase conjugate (#170–6515) and goat anti-mouse IgG-horseradish peroxidase conjugate (#170–6516) were from Bio-Rad (Hercules, CA, U.S.).

### 2.8 Statistics

Data are presented as means with the standard error of the mean (± SEM). In all experiments, n refers to the number of donors (primary cultures obtained from different donors). Statistical analysis was performed with Microsoft Excel (Microsoft Office 2016) and GraphPad Prism 6 (GraphPad Software). Statistical significance was determined using t-test or a two-way ANOVA with Tukey *post hoc* test. The difference between the groups was considered statistically significant when p ≤ 0.05.

## 3 Results

### 3.1 Expression of PNPLA7 in cultured human skeletal muscle cells

**Figure 1:**
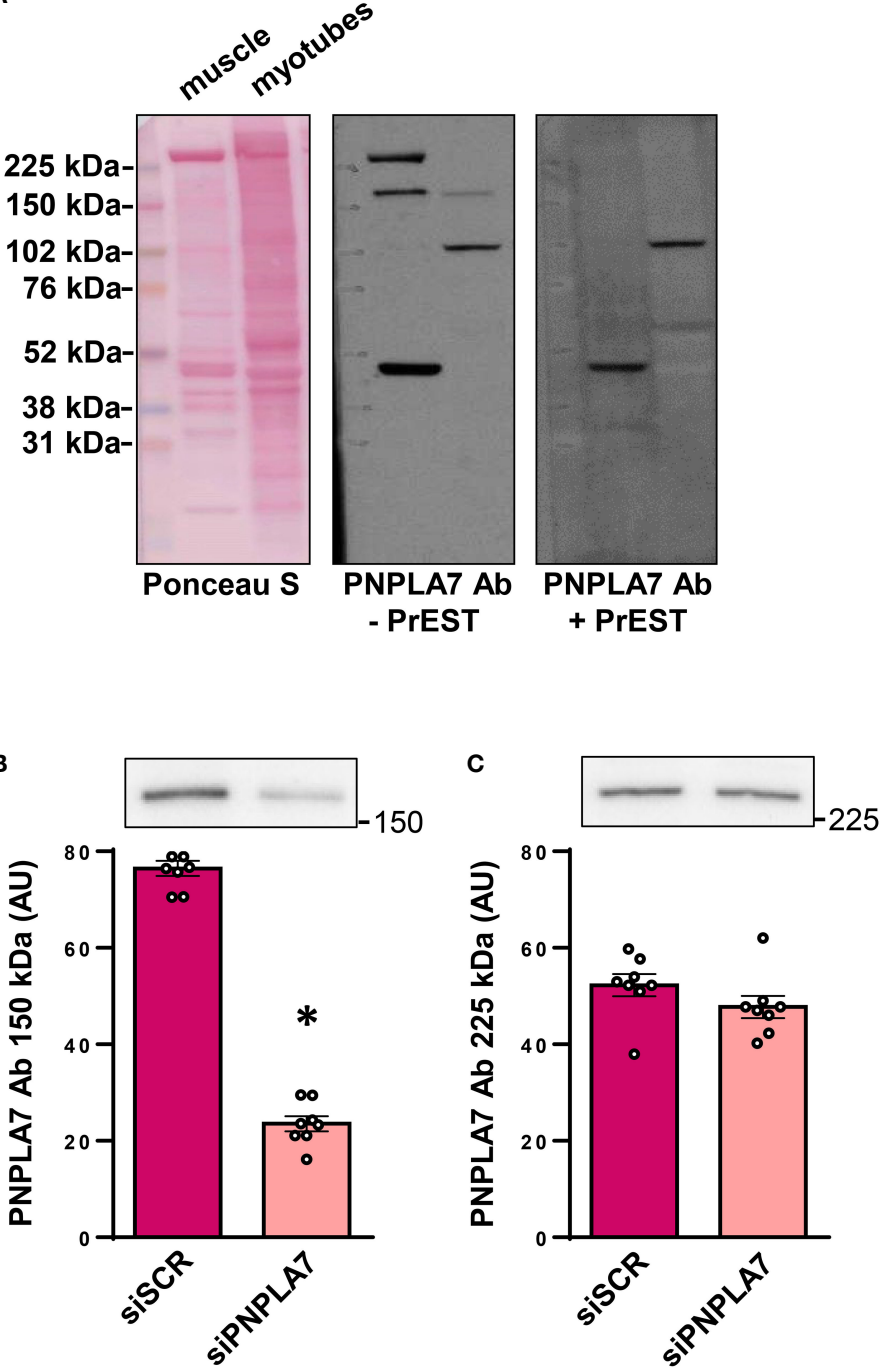
Expression of PNPLA7 in cultured human skeletal muscle cells: **(A)** Immunoblot of human *semitendinosus* muscle sample and human myotube sample (differentiated for 10 days), stained by Ponceau S, immunostained with PNPLA7 antibody (- PrEST) or immunostained with neutralized PNPLA7 antibody, prepared by preincubation with its own antigen peptid (+ PrEST). **(B, C)** Gene silencing: myoblasts were treated with siRNA against *PNPLA7* mRNA (siPNPLA7) or scrambled siRNA (siSCR). Densitometry of immunoreactive bands **(B)** at ~150 kDa and **(C)** ~225 kDa was performed 72h after the addition of siRNA. Results are means with SEM (n = 7). *p < 0.05 vs. siSCR.

The nutritional status-dependent expression of *Pnpla7* mRNA in mouse skeletal muscle was determined using Northern blot (8). To establish whether human skeletal muscle cells functionally express PNPLA7, we performed immunoblot using anti-PNPLA7 from Sigma/Merck (HPA009130) (9), which produced several immunoreactive bands in human skeletal muscle tissue and cultured myotubes, including a band at the predicted molecular weight (~150 kDa) (**Figure 1A**), consistent with our preliminary observations (28). To validate the result, HPA009130 was neutralized with PrEST antigen PNPLA7 (APREST71921). Upon incubation with the neutralized antibody, bands at ~150 and ~225 kDa disappeared, supporting the notion that the ~150 kDa band corresponds to PNPLA7. To independently verify this interpretation, gene silencing was performed using cultured human myoblasts and siRNA against PNPLA7 (**Figures 1B, C**). Treatment with siRNA reduced the intensity of the ~150 kDa band (**Figure 1B**), but not the ~225 kDa band (**Figure 1C**), consistent with the notion that the ~150 kDa band represents PNPLA7.

As an additional control, the antigen sequence of HPA009130 was tested for possible alignments with other proteins using BLAST (36), which revealed 100% alignment with PNPLA7 and 42.86% alignment with PNPLA6 (https://blast.ncbi.nlm.nih.gov/Blast.cgi#sort_mark, accessed on the 9^th^ September 2022). Taken together, these results indicated that primary human skeletal muscle cells express PNPLA7 protein and that the ~150 kDa band, obtained with HPA009130 could be used for its quantification.

### 3.2 Functional assessment of PNPLA7 in cultured human myoblasts

**Figure 2:**
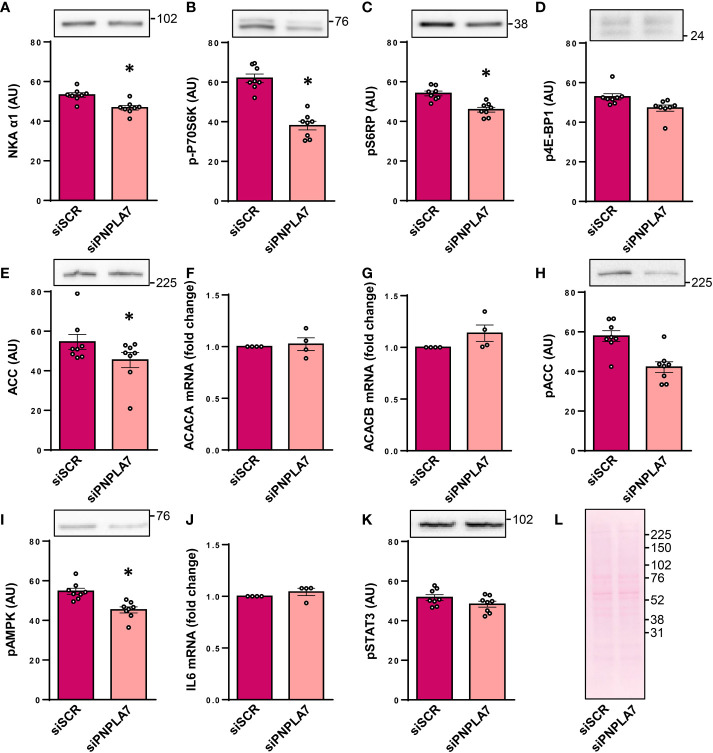
Functional assessment of PNPLA7 in cultured human myoblasts. Myoblasts were treated with siRNA against *PNPLA7* mRNA (siPNPLA7) or scrambled siRNA (siSCR). Functional consequences of PNPLA7 knock-down **(A–K)** were estimated by measuring **(A)** the abundance of α1-subunit of Na^+^,K^+^-ATPase (NKAα1), **(B)** phosphorylation of p70S6K^Thr389^, **(C)** phosphorylation of S6RP^Ser235/236^, **(D)** phosphorylation of 4E-BP1 Thr^37/46^, **(E)** the abundance of ACC, **(F)** *ACC1* mRNA (gene *ACACA*), **(G)** *ACC2* mRNA (gene *ACACB*), **(H)** phosphorylation of ACC^Ser79^, **(I)** phosphorylation of AMPK^Thr172^, **(J)** *IL6* mRNA, and **(K)** phosphorylation of STAT3^Tyr705^. Ponceau S staining was used as a control of loading and transfer of immunoblotting **(A–E, H–K)**. **(L)** A representative Ponceau-stained membrane. Actin blots, which were used as an additional loading control are shown in the corresponding **Supplementary Figure**. The mRNA levels of IL-6, ACC1, and ACC2, which were measured using qPCR, are presented as geometric mean of expression ratios against two reference genes, 18S rRNA and ACTB. Results are means with SEM (n = 7). *p < 0.05 vs. siSCR.

The widely expressed catalytic α1-subunit of Na^+^,K^+^-ATPase (NKA aka sodium pump), whose function is known to be affected by lysophosphatidylcholine and membrane lipid environment (37, 38), was reduced by knocking-down PNPLA7 (**Figure 2A**), indicating deficiency of PNPLA7 had functional consequences for human skeletal muscle cells.

In mice, *Pnpla7* was upregulated in skeletal muscle during fasting (8), which indirectly suggested that it might functionally interact with nutrient- and energy status-sensitive pathways, such as the mTOR pathway and the AMP-activated protein kinase (AMPK) pathway. The mTOR pathway was assessed by measuring the phosphorylation of 70kDa ribosomal protein S6 kinase at Thr^389^ (p70S6K, **Figure 2B**), an activating phosphorylation catalysed by mTOR complex 1 (mTORC1), and phosphorylation of its substrate ribosomal protein S6 (S6RP) at Ser^235/236^ (**Figure 2C**). The phosphorylation of 4E-BP1 at Thr^37/46^ (**Figure 2D**), another direct substrate of mTORC1, was also measured. Gene silencing of *PNPLA7* reduced the phosphorylation of p70S6K and ribosomal protein S6, but not that of 4E-BP1, which suggests that while PNPLA7 may modulate the mTOR pathway it exerts divergent effects on its downstream effectors.

The AMPK pathway was assessed by measuring the expression and phosphorylation of AMPK substrate acetyl-CoA carboxylase (ACC), which plays a major role in fatty acid metabolism by catalyzing the conversion of acetyl-CoA to malonyl-CoA. The abundance of the total ACC was reduced in PNPLA7 knock-down cells (**Figure 2E**). This was apparently not a transcriptional effect since mRNA levels of the ACC1 isoform (aka ACCα, gene *ACACA*), which by producing malonyl-CoA catalyzes the first step of *de novo* synthesis of fatty acids, and the ACC2 isoform (aka ACCβ, gene *ACACB*), which by producing malonyl-CoA inhibits carnitine palmitoyltransferase and β-oxidation, were similar in control and knock-down cells (**Figures 2F, G**). The phosphorylation of ACC at Ser^79^, the AMPK-sensitive site, tended to be lower in PNPLA7 knock-down cells (**Figure 2H**) but the difference did not reach the level of statistical significance. The level of phosphorylated (*i.e.* active) AMPK (at Thr^172^) was significantly reduced (**Figure 2I**), consistent with the apparent reduction in the phosphorylated ACC in knock-down cells.

*Pnpla7* knock-out mice demonstrated prominent myopathic changes, increased plasma levels of interleukin-6 (IL-6) (27), and low hepatic *Il6* mRNA expression (19). To determine whether gene silencing of *PNPLA7* might modulate IL-6 signalling in human skeletal muscle cells, which robustly express IL-6 and STAT3, a transcriptional factor through which IL-6 triggers its effects (31, 39, 40), we measured *IL6* mRNA and the phosphorylation (Tyr^705^) of STAT3. The expression of *IL6* mRNA (**Figure 2J**) and the abundance of phospho-STAT3 (**Figure 2K**) were similar in control and knock-down cells, indicating PNPLA7 does not regulate IL-6 secretion and action under basal conditions.

Collectively, these results suggested that deficiency of PNPLA7 selectively affects various aspects of myoblast function, thereby indirectly indicating a role for PNPLA7 in human skeletal muscle.

### 3.3 Effect of insulin, dexamethasone, forskolin, and glucose on the PNPLA7 expression in cultured human myotubes

**Figure 3:**
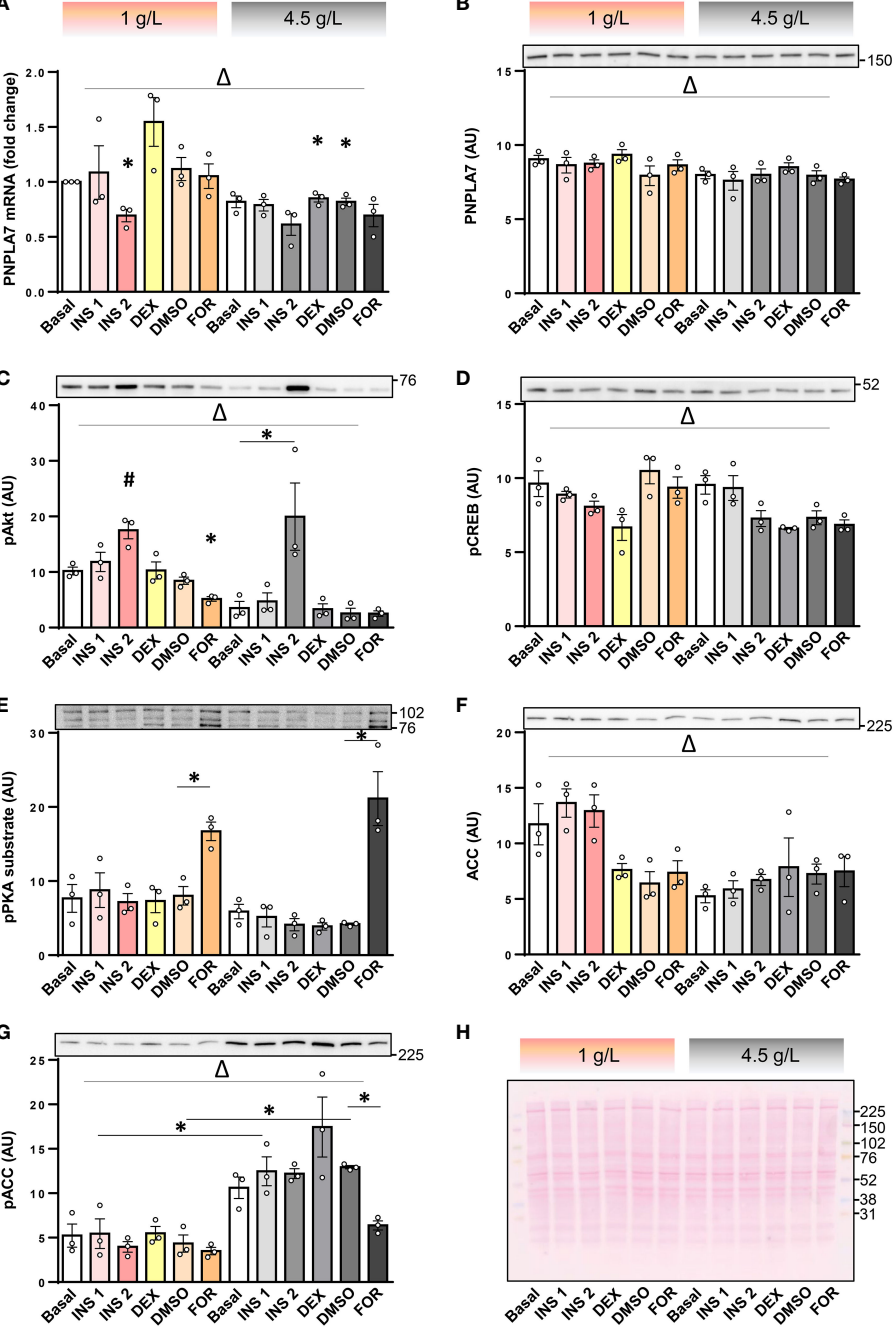
Effect of insulin, dexamethasone, forskolin, and glucose on the PNPLA7 expression in cultured human myotubes. **(A–G)** Human skeletal muscle cells were differentiated for 8-10 days in Advanced MEM with 2% FBS. For the last 16 h of the experiment cells were grown in the serum-free DMEM with two different glucose concentrations (1 g/L and 4.5 g/L; marked at the top with pink and grey colour code, respectively), and exposed to insulin (0.1 µg/mL; INS1, or 10 μg/mL; INS2), dexamethasone (1 μM; DEX), or forskolin (5 µM in DMSO; FOR), or DMSO (0.05 v/v %). **(A)** The mRNA levels of PNPLA7 were measured by qPCR and are presented as geometric mean of expression ratios against two reference genes (18S rRNA and ACTB). **(B–G)** Protein levels of **(B)** PNPLA7, **(C)** phospho-Akt Ser^473^, **(D)** phospho-CREB Ser^133^, **(E)** phospho-PKA substrate, **(F)** ACC, and **(G)** phospho-ACC Ser^79^, were estimated by immunoblotting (representative blots are shown above the charts with indicated positions of molecular weight markers; kDa). **(H)** A representative Ponceau-stained membrane. Actin blots, which were used as an additional loading control are shown in the corresponding **Supplementary Figure**. Results are means with SEM (n = 3). *p < 0.05 vs. Basal-1 g/L glucose or as indicated. ^Δ^ p < 0.05 between 1 g/L and 4.5 g/L glucose (two-way ANOVA), #Dunnett test vs. Basal.

Feeding reduced *Pnpla7* mRNA levels in mouse skeletal muscle (8), suggesting its expression might be regulated by metabolic hormones or nutritional factors. Here we examined whether insulin or its counterregulatory hormones may regulate expression of PNPLA7 in human skeletal muscle cells. To examine this question, cultured human myotubes were treated with 0.1 or 10 µg/mL insulin, 1 μM dexamethasone (a synthetic glucocorticoid), and 5 μM forskolin (to mimic activation of adenylyl cyclase by catecholamines). Experiments were carried out in serum-free DMEM with physiological (1 g/L ≈ 5.6 mM) or high (4.5 g/L = 25 mM) concentration of glucose. At 1 g/L glucose, 10 μg/mL insulin reduced mRNA levels of PNPLA7 (**Figure 3A**), but had no effect on its protein abundance (**Figure 3B**). At 4.5 g/L glucose, insulin had a similar effect on *PNPLA7* mRNA, but the difference did not reach statistical significance. PNPLA7 levels were not significantly altered by dexamethasone and forskolin. Although PNPLA7 protein was unresponsive to pharmacological treatments, two-way ANOVA showed that both its mRNA and protein levels were significantly different between normal and high glucose conditions. The phosphorylation of Akt, a major kinase downstream of insulin receptor, was increased more prominently at 4.5 g/L glucose (**Figure 3C**), suggesting that the extent to which insulin signalling pathway was activated did not directly correlate with the effect of insulin on *PNPLA7* mRNA levels. Phosphorylation of cAMP response element-binding protein (CREB), a well-characterized down-stream target of PKA (**Figure 3D**), was not increased by forskolin, but as assessed by anti-PKA substrate antibody, PKA was effectively activated in this experiment (**Figure 3E**), suggesting CREB might not be a good marker of forskolin-induced PKA activation within this timeframe. The abundance of ACC was lower at 4.5 g/L glucose than at 1 g/L glucose (**Figure 3F**), while inhibitory phosphorylation of ACC at Ser^79^ showed an opposite response (**Figure 3G**).

### 3.4 Effect of insulin, dibutyryl-cAMP, and glucose on the expression of PNPLA7 in cultured myotubes

**Figure 4:**
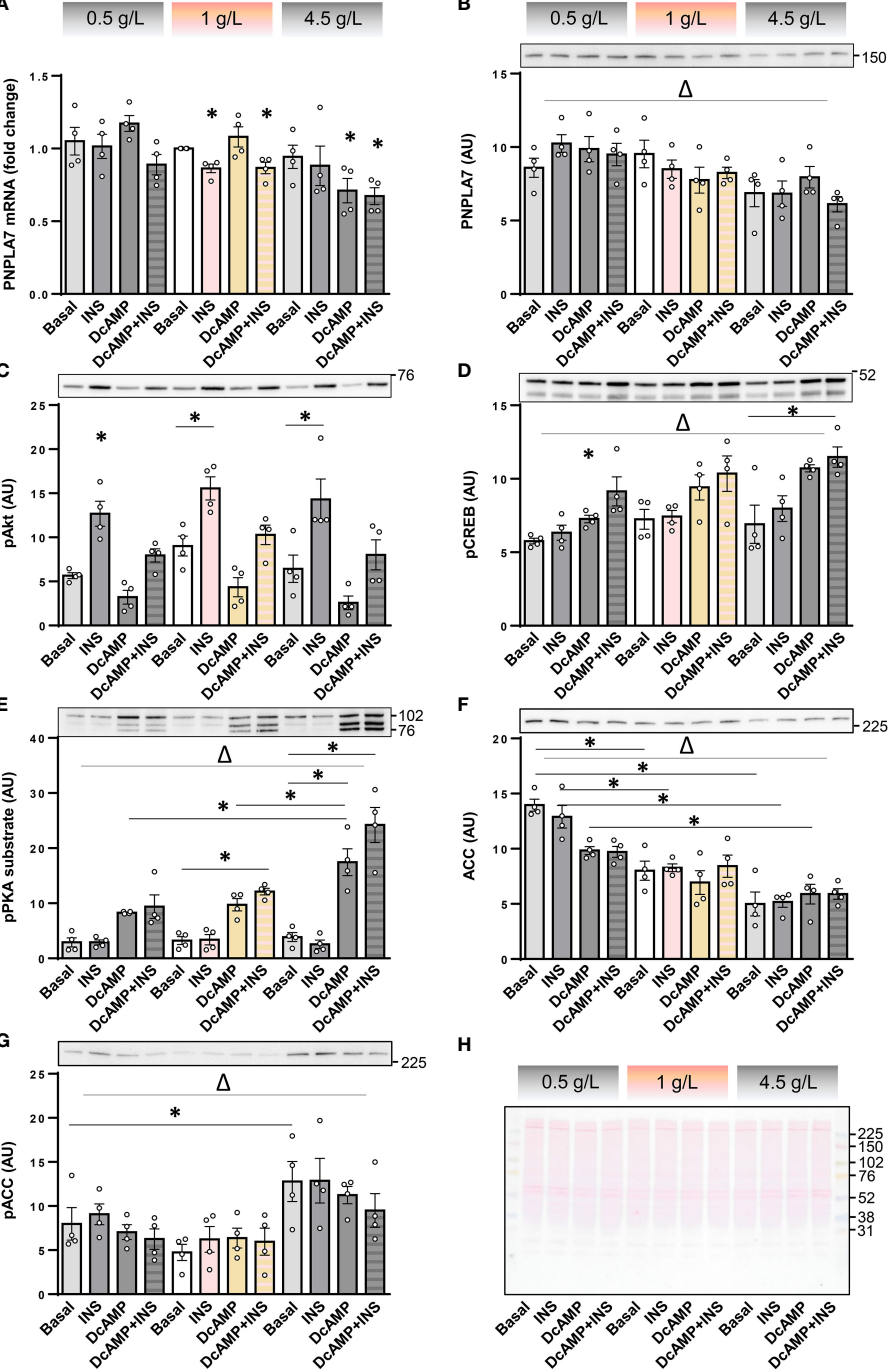
Effect of insulin, dibutyryl-cAMP, and glucose on the expression of PNPLA7 in cultured myotubes. **(A–G)** Human skeletal muscle cells were differentiated for 8-10 days in Advanced MEM with 2% FBS. For the last 16 h of the experiment cells were grown in serum starvation DMEM with low (0.5 g/L), normal (1 g/L), or high glucose concentration (4.5 g/L) (marked at the top with the grey-pink-grey colour code), and exposed at the same time to insulin (0.1 µg/mL; INS) and/or dibutyryl-cAMP (200 µM; DcAMP), or vehicle (Basal). **(A)** Transcript levels of PNPLA7 were measured by qPCR and are presented as geometric mean of expression ratios against two reference genes, 18S rRNA and ACTB. **(B–G)** Protein levels of **(B)** PNPLA7 and markers of activity of metabolic pathways/metabolic status, **(C)** phospho-Akt Ser^473^, **(D)** phospho-CREB Ser^133^, **(E)** phospho-PKA substrate, **(F)** ACC, and **(G)** phospho-ACC Ser^79^, were assessed by immunoblotting. **(H)** A representative Ponceau-stained membrane. Actin blots, which were used as an additional loading control are shown in the corresponding **Supplementary Figure**. Results are means with SEM (n = 4, including one donor from Sigma-Aldrich, Merck). *p < 0.05 vs. Basal-0.5 g/L glucose or as indicated. ^Δ^ p < 0.05 two-way ANOVA between different glucose concentrations.

Counter-regulatory hormones, such as catecholamines, are particularly important for regulation of metabolic homeostasis during hypoglycaemia, which may occur due to insulin or insulin unrelated causes. To determine whether effects of insulin and PKA activation are modulated by low glucose concentrations, cultured human myotubes were exposed to insulin and/or dibutyryl-cAMP (db-cAMP), a cell-permeable analogue of cAMP, at low (0.5 g/L ≈ 2.78 mM), physiological (1 g/L), or high (4.5 g/L) glucose concentrations. The *PNPLA7* mRNA levels tended to decrease with increasing glucose concentrations (**Figure 4A**), which was paralleled by a significant decrease in the protein abundance (**Figure 4B**). Insulin again reduced *PNPLA7* mRNA levels at 1 g/L glucose (**Figure 4A**) without affecting its protein abundance (**Figure 4B**). Insulin had no effect on PNPLA7 at 0.5 and 4.5 g/L glucose, although the phosphorylation of Akt (**Figure 4C**) was increased by insulin at all glucose concentrations. Db-cAMP reduced the expression of *PNPLA7* mRNA only at 4.5 g/L, while it increased or tended to increase the phosphorylation of CREB (**Figure 4D**) as well as PKA substrates (**Figure 4E**) at all glucose concentrations. The abundance of ACC increased in parallel with decreasing glucose concentrations, reaching the highest levels at 0.5 g/L glucose (**Figure 4F**). Conversely, the phosphorylation of ACC was most pronounced at 4.5 g/L glucose (**Figure 4G**).

## 4 Discussion

Studies concerning the role of PNPLA7 are rare and only a couple of them investigated PNPLA7 in skeletal muscle (8, 19, 27). Importantly, mouse studies indicate that expression of *PNPLA7* mRNA in skeletal muscle is dependent on hormonal or metabolic status as well as that PNPLA7 deficiency leads to myopathy, highlighting the functional significance of PNPLA7 in skeletal muscle physiology. However, little is known about the expression and regulation of PNPLA7 in human skeletal muscle. Our preliminary results suggested that human myotubes might express PNPLA7 protein (28), but this result required further validation, which we provide in the current study. Here, we demonstrate that cultured human myoblasts and myotubes express *PNPLA7* mRNA as well as PNPLA7 protein. This result is consistent with data reported on Skeletal Muscle RNAlysis site, which indicates PNPLA7 expression in primary human skeletal muscle cells as well as mouse C2C12 and rat L6 cells. Importantly, we show that gene silencing of PNPLA7 reduced the activity of the mTOR pathway as well as the abundance of α1-subunit of Na^+^,K^+^-ATPase, which indirectly suggests that PNPLA7 is not only expressed, but is also functionally important for human skeletal muscle cells.

As assessed by measuring the phosphorylation of p70S6K and ribosomal protein S6, deficiency of PNPLA7 suppresses mTOR signalling. Once phosphorylated (activated) by mTORC1, p70S6K phosphorylates ribosomal protein S6, thus promoting protein translation (41, 42). While the molecular mechanisms that underlie this link are unknown, lack of PNPLA7, which is normally located in the membrane of endoplasmic reticulum, may lead to disruption of its membranes, thus inducing endoplasmic reticulum stress (41). However, phosphorylation of 4E-BP1, a repressor of protein translation, was unaltered in PNPLA7-deficient myoblast, indicating effectors downstream of mTORC1 are regulated in a distinct manner.

In mice, fasting increases, while feeding decreases *Pnpla7* mRNA levels in skeletal muscle (8). The mRNA expression of PNPLA7 in cultured mouse adipocytes is decreased by insulin (8), indirectly suggesting that fluctuations in insulin concentrations may underlie feeding-dependent suppression of *Pnpla7* expression. In the current study, we determined that the expression of *PNPLA7* mRNA, but not protein abundance, was suppressed by insulin at physiological glucose concentrations. Since we did not determine the half-life of PNPLA7 protein in cultured myotubes it is difficult to judge whether the lack of correspondence between mRNA and protein levels is due to divergent regulatory mechanisms or whether the timeframe (16 hours) was not sufficient to observe a decrease in PNPLA7 protein levels. Interestingly, insulin did not alter *PNPLA7* mRNA levels at low (0.5 g/L) or high (4.5 g/L) glucose, showing that glucose modulated its expression. Consistent with this view, the protein abundance of PNPLA7 in cultured myotubes inversely correlated with glucose concentrations. Taken together with previously published work (8), this result suggests a model whereby a postprandial increase in glycaemia and/or insulinaemia suppresses *PNPLA7* expression in skeletal muscle, while their postabsorptive decrease may induce it.

In the fasted state, counterregulatory hormones contribute to regulation of metabolic homeostasis. We therefore examined whether dexamethasone or activation of the PKA pathway (to mimic activation of β-adrenergic receptors by catecholamines) modulates expression of *PNPLA7* in human myotubes. While dexamethasone tended to increase *PNPLA7* mRNA at 1 g/L glucose, the effect was not statistically significant and was not observed at 4.5 g/L glucose. Moreover, dexamethasone had no effect on the PNPLA7 protein abundance. Clearly, these results do not support the notion that glucocorticoids may play a prominent role in regulation of PNPLA7. Activation of PKA by forskolin, an adenylyl cyclase activator, also did not alter PNPLA7 levels. Conversely, db-cAMP reduced the *PNPLA7* mRNA levels at 4.5 g/L glucose, indicating that the cAMP/PKA pathway may exert negative control over PNPLA7 expression. Interestingly, it was previously shown that PNPLA6, a PNPLA7 homologue, is upregulated by cAMP/PKA in HeLa cells (43). Furthermore, CREB was shown to bind to the *PNPLA6* promoter and to upregulate its expression through cAMP/PKA signalling pathway (44). It would be important to determine whether db-cAMP suppresses PNPLA7, which contains three cyclic nucleotide binding domains (9, 45, 46), *via* PKA/CREB or *via* direct binding to PNPLA7. While cAMP binding *per s*e does not affect enzymatic activities of either PNPLA6 or PNPLA7 (1, 47), there is an interesting possibility that cAMP affects the subcellular localization of PNPLA7 in skeletal muscle cells. For instance, PNPLA7 in non-muscle cells was shown to associate with lipid droplets after stimulation with cyclic nucleotides (9, 10).

Gene silencing of *PNPLA7* did not affect expression level of *IL6* mRNA or phosphorylation of STAT3, indicating PNPLA7 is not important for IL-6 signalling in human skeletal muscle cells. Conversely, in *Pnpla7* knock-out mice the concentrations of IL-6 in plasma were increased (27), while the expression of *Il6* and *Il1b* mRNA expression in the liver was decreased (19). These alterations in cytokine secretion and expression might be secondary to skeletal muscle dysfunction and local or systemic metabolic alterations, which were observed in the murine knock-out model. This could explain why there was no effect on *IL6* expression or action in our experiments. However, the expression of *Pnpla7* in cultured murine macrophages was reduced by bacterial lipopolysaccharide (48). Moreover, overexpression of *Pnpla7* suppressed and gene silencing of *Pnpla7* enhanced lipopolysaccharide-induced expression of proinflammatory cytokines, such as IL-1β (gene *Il1b*) (48). While these results suggest a direct role for PNPLA7 in regulation of cytokine secretion from murine macrophages, it would not be unreasonable to suggest that secretion of IL-6 from human skeletal muscle cells is regulated in a different manner. However, the possibility that the extent of knock-down (a ~75% reduction) in our experiments was not sufficient to affect expression or action of IL-6 should also be considered.

The interpretation of our results concerning the expression of *PNPLA7* depends on the interpretation of the immunoblotting data, which might be affected by non-specific binding of the primary antibody HPA009130. To avoid technical artefacts, we performed antibody validation experiments, which included antibody neutralization and gene silencing of *PNPLA7*. Antibody neutralization experiments indicated that both the ~150 kDa and ~225 kDa likely reflect specific binding of HPA009130. Taken together with the predicted molecular weight of PNPLA7 (~145.7 kDa) and gene silencing experiments, which resulted in marked suppression of the ~150 kDa band, it is highly likely that the ~150 kDa band represents PNPLA7. Conversely, the ~225 kDa band, which was not detectable during incubation with PrEST-treated HPA009130, was not affected by gene silencing, suggesting it does not represent a protein encoded by the *PNPLA7* gene. The discrepancy could be explained if a dimer or another PNPLA7-containing complex has a longer half-life than a PNPLA7 monomer, meaning that incubation was too short to observe a significant decrease in the ~225 kDa immunoreactivity. However, while patatin, isolated from *Solanum tuberosum*, is expressed as a dimer (49), a PNPLA7 dimer would be expected to run at higher molecular weights. Moreover, the patatin dimer dissociates in the SDS-containing media (49), meaning that any potential dimer likely decomposed during sample preparation in Laemmli buffer, which contains high concentrations of SDS. Recently, the crystal structure of PNPLA9, the only PNPLA enzyme whose structure has been resolved so far, showed that PNPLA9 crystallizes as a functional dimer (50), which might be resolved close to the 225 kDa immunoreactive band. However, antibody neutralization experiment as well as BLAST, which showed that recombinant protein fragment, which had been used for production of the HPA009130, did not align, do not support the notion that the ~225 kDa band represents cross-reactivity with PNPLA9. Clearly, the identity of the ~225 kDa band needs to be confirmed in future studies.

Primary human skeletal muscle cells are a standard model for skeletal muscle research under *in vitro* conditions (51). One of the advantages of using this model is that these cells retain at least some of the characteristics of the human donors of skeletal muscle tissue (52, 53). However, on the other hand, primary cultures, which also contain non-muscle cells, are not as homogenous as cultures of animal cell lines, such as L6 or C2C12 cells. Donor variability and variable cellular composition of the primary culture contribute to variability of experimental results, which are typically more scattered than those that are obtained from cell lines. For instance, cells from different donors may have markedly different basal expression or phosphorylation levels of various target proteins (29, 31). The fold-response to different stimuli may also markedly differ (31), which may pose a challenge for statistical analysis and subsequent data interpretation. Higher variability cannot be always counterbalanced by including more independent cultures and experiments, their number being limited both by the number of available donors and limited proliferative capacity of primary human skeletal muscle cells, which reduces the number of passages that can be performed. Nevertheless, animal cell lines, although undoubtedly useful for many applications (51), do not always represent a suitable replacement because differences in expression patterns and functional characteristics of target proteins may lead to species-specific responses (31), thus reducing the possibility of translation. Taken together, despite the disadvantages, which must always be taken into consideration, primary human skeletal muscle cells represent a highly useful model to study molecular physiology and pathophysiology of human skeletal muscle.

## 5 Conclusions

1. Primary human skeletal muscle cells express *PNPLA7* mRNA and protein.
2. The abundance of the α1-subunit of Na^+^, K^+^-ATPase, the phosphorylated p70S6K, the phosphorylated ribosomal protein S6, and ACC are reduced in PNPLA7-deficient human myoblasts, suggesting PNPLA7 is functionally important for these cells.
3. Expression of *PNPLA7* mRNA in cultured human myotubes is suppressed by insulin at 1 g/L glucose and db-cAMP at 4.5 g/L glucose.
4. The protein abundance of PNPLA7 in cultured human myotubes correlates inversely with the glucose concentration.
5. Collectively, our results implicate a role for PNPLA7 in skeletal muscle energy metabolism.

## Data availability statement

The original contributions presented in the study are included in the article/**Supplementary Material**. Further inquiries can be directed to the corresponding authors.

## Ethics statement

The studies involving human participants were reviewed and approved by the Republic of Slovenia National Medical Ethics Committee (ethical approval no. 71/05/12 and 0120-698/2017/4). Full statement: The primary human skeletal muscle cell cultures were prepared from the samples of semitendinosus muscle obtained during routine orthopaedic surgery as surgical waste, with informed written consent signed by the participants as described (28–31). Preparation of primary human skeletal muscle cells and experimental procedures involving these cells were approved by the Republic of Slovenia National Medical Ethics Committee (ethical approval no. 71/05/12 and 0120-698/2017/4). The patients/participants provided their written informed consent to participate in this study.

## Author contributions

KM and AM-L performed experiments. MK, AM-L, and KM analyzed the data and prepared the first draft of the manuscript. TM planned experiments and analyzed the data. SP analyzed the data, edited, redrafted, and finalized the manuscript. SP and MK provided funds, planned and supervised the experiments. SP, KM, and MK conceptualized the study. All authors contributed to the article and approved the submitted version.
