# Supplementary material for: Insulin, dibutyryl-cAMP, and glucose modulate expression of patatin-like domain containing protein 7 in cultured human myotubes
Source: Front Endocrinol (Lausanne). 2023 Mar 22;14:1139303. doi: 10.3389/fendo.2023.1139303 (PMC10073714; doi:10.3389/fendo.2023.1139303)
Supplement: Supplementary file 1 [file Image_1.pdf]

Insulin, dibutyryl-cAMP, and glucose modulate expression of patatin-like domain containing protein 7 in cultured human myotubes

Katarina Miš[1], Ana-Marija Lulić[2], Tomaž Marš[1], Sergej Pirkmajer[1,*,#], and Maja Katalinić[2,*,#]

[1]Institute of Pathophysiology, Faculty of Medicine, University of Ljubljana, SI-1000 Ljubljana, Slovenia

[2]Biochemistry and Organic Analytical Chemistry Unit, Institute for Medical Research and Occupational Health, HR-10000 Zagreb, Croatia

*These authors contributed equally to this work and share senior authorship.

#Correspondence:

Sergej Pirkmajer, MD, PhD

sergej.pirkmajer@mf.uni-lj.si

Maja Katalinić, PhD

mkatalinic@imi.hr

Figure 1

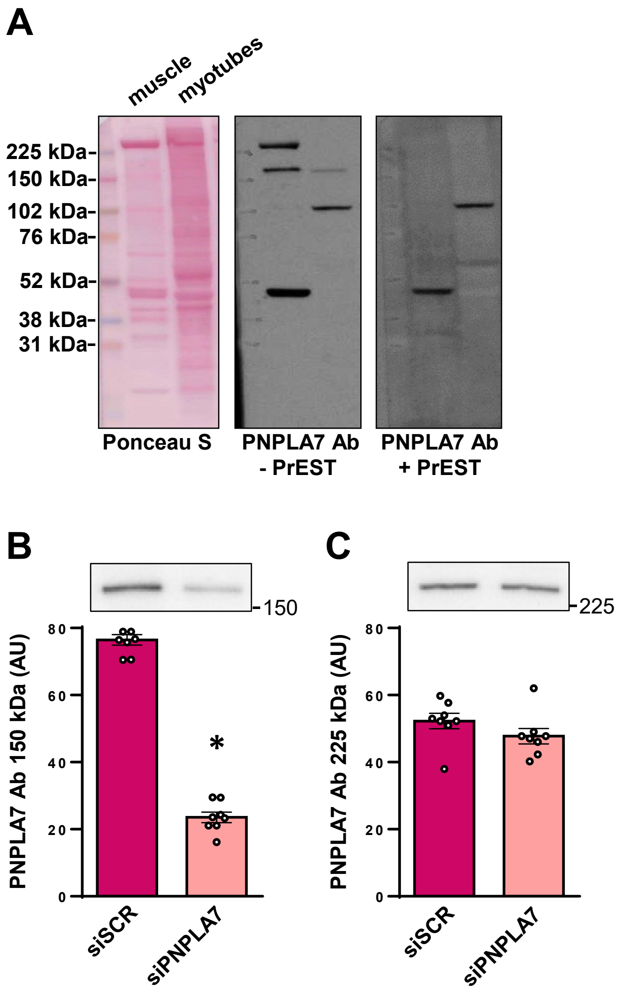
A
muscle
myotubes
225 kDa-
150 kDa-
102 kDa-
76 kDa-
52 kDa-
38 kDa-
31 kDa-
Ponceau S
PNPLA7 Ab
- PrEST
PNPLA7 Ab
+ PrEST
B
150
PNPLA7 Ab 150 kDa (AU)
80
60
40
20
0
*
siSCR
siPNPLA7
C
225
PNPLA7 Ab 225 kDa (AU)
80
60
40
20
0
siSCR
siPNPLA7

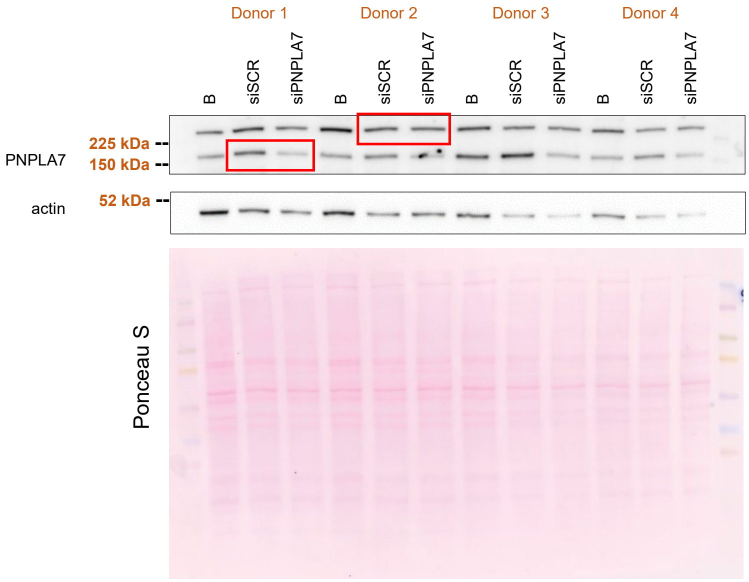

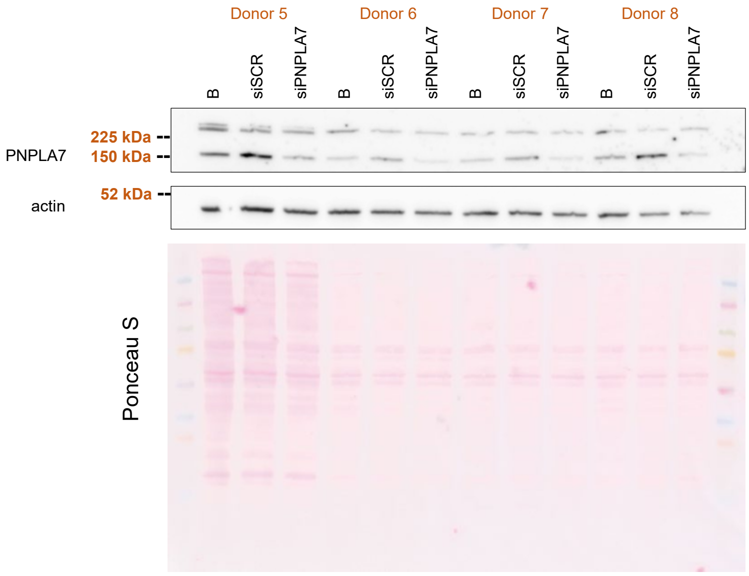

The frame shows the blots that are presented in the figure.

Figure 2

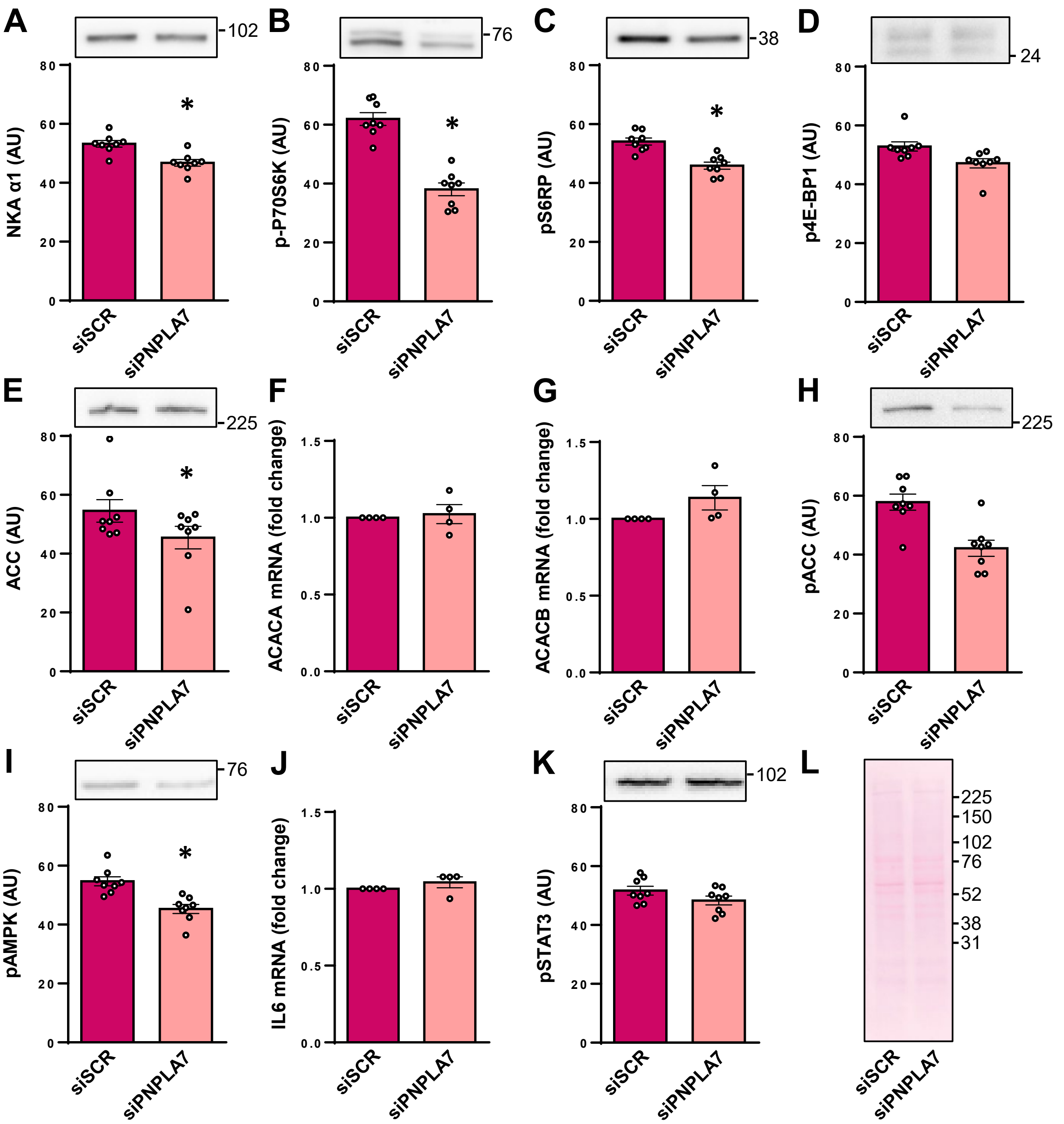

A
102
NKA α1 (AU)
B
76
p-P70S6K (AU)
C
38
pS6RP (AU)
D
24
p4E-BP1 (AU)
E
225
ACC (AU)
F
ACACA mRNA (fold change)
G
ACACB mRNA (fold change)
H
225
pACC (AU)
I
76
pAMPK (AU)
J
IL6 mRNA (fold change)
K
102
pSTAT3 (AU)
L
225
150
102
76
52
38
31
siSCR
siPNPLA7
*

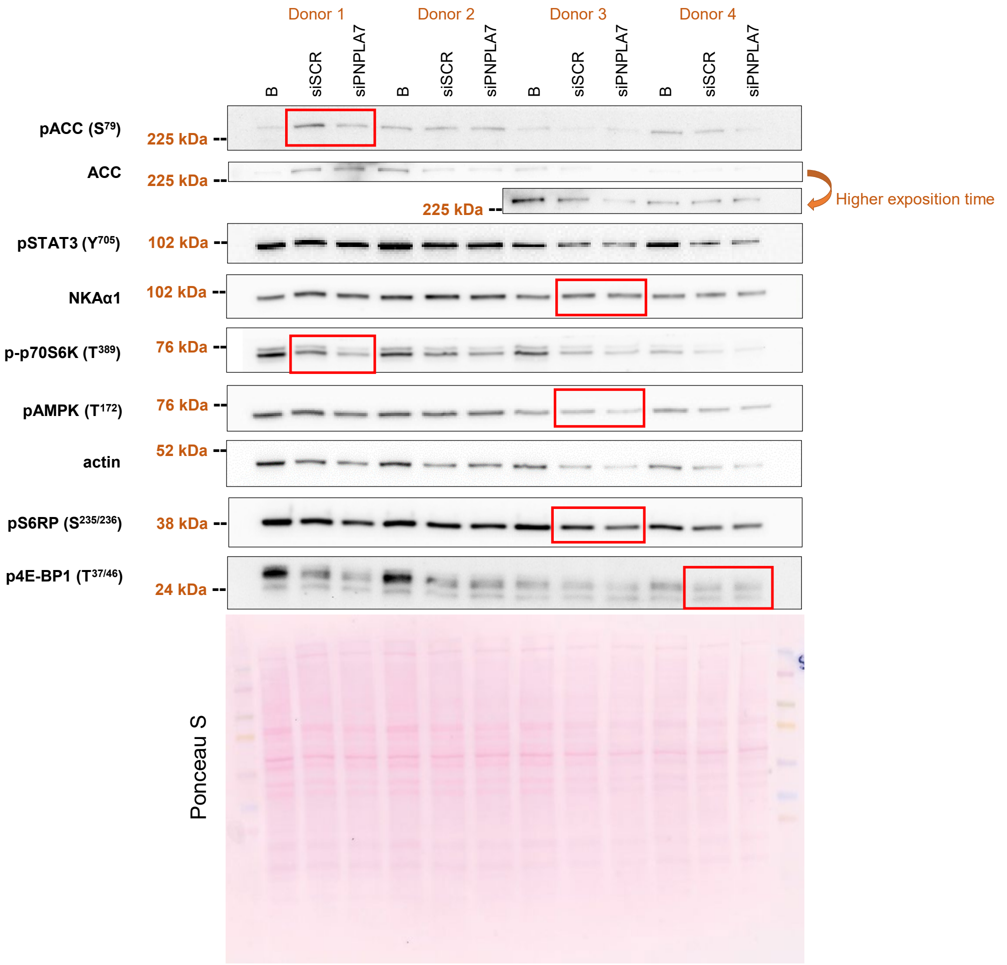

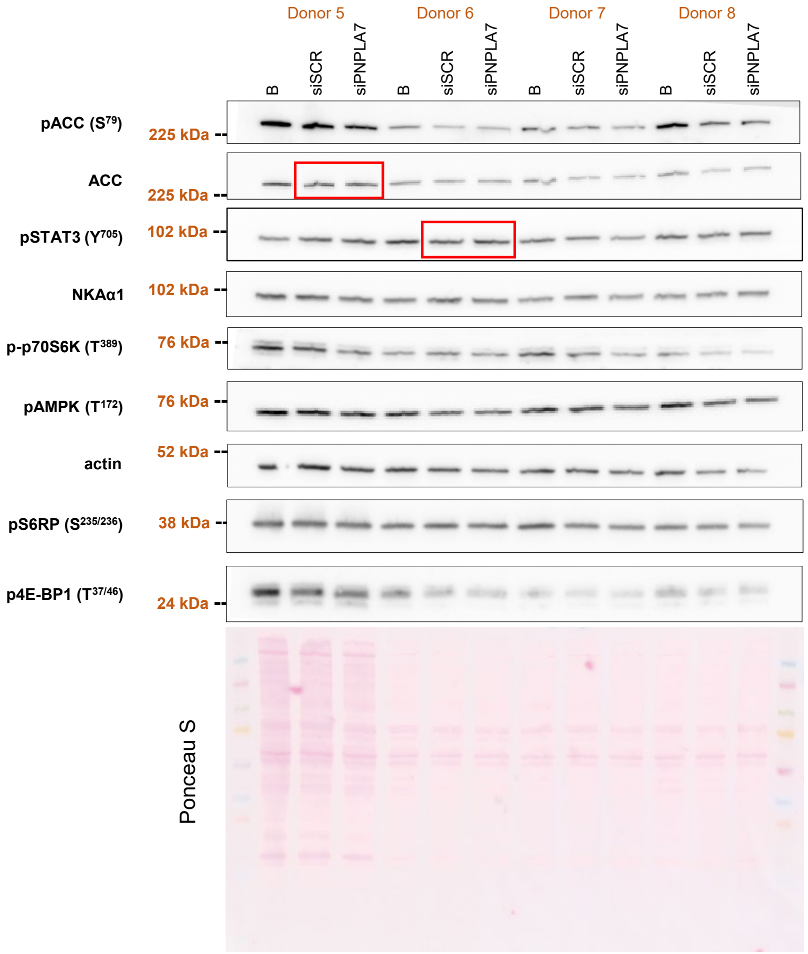

The frame shows the blots that are presented in the figure.

Figure 3

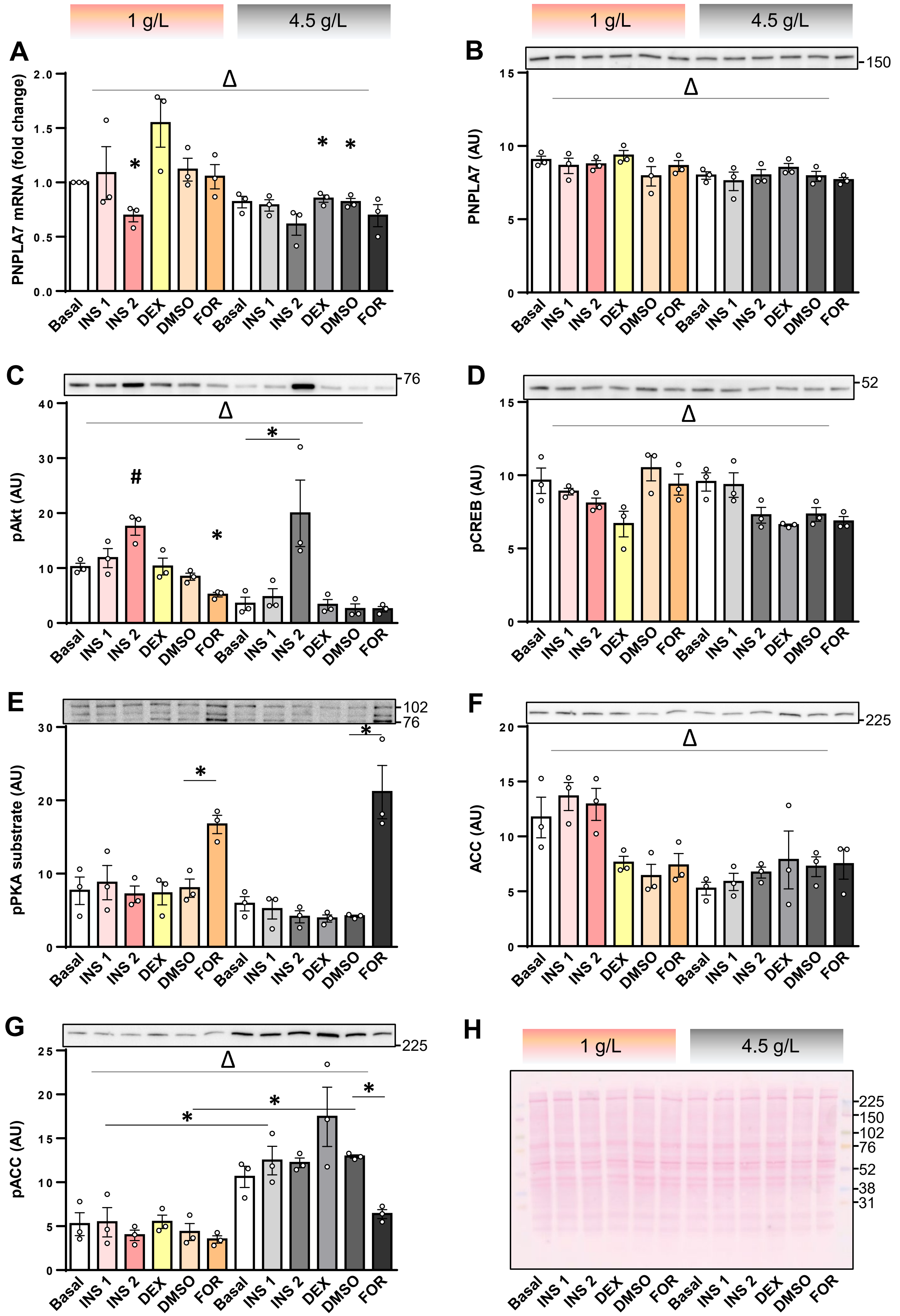

1 g/L
4.5 g/L
A
PNPLA7 mRNA (fold change)
B
PNPLA7 (AU)
150
C
pAkt (AU)
76
D
pCREB (AU)
52
E
pPKA substrate (AU)
102
76
F
ACC (AU)
225
G
pACC (AU)
225
H
225
150
102
76
52
38
31
Basal
INS 1
INS 2
DEX
DMSO
FOR
Basal
INS 1
INS 2
DEX
DMSO
FOR

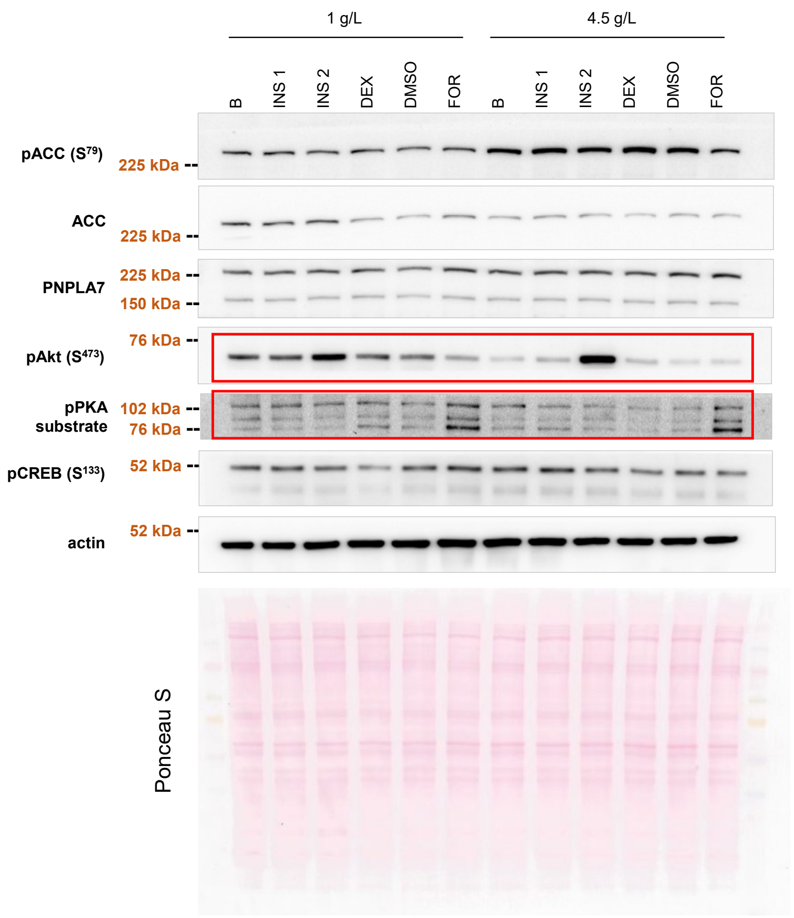

Donor 1 (IDF4)

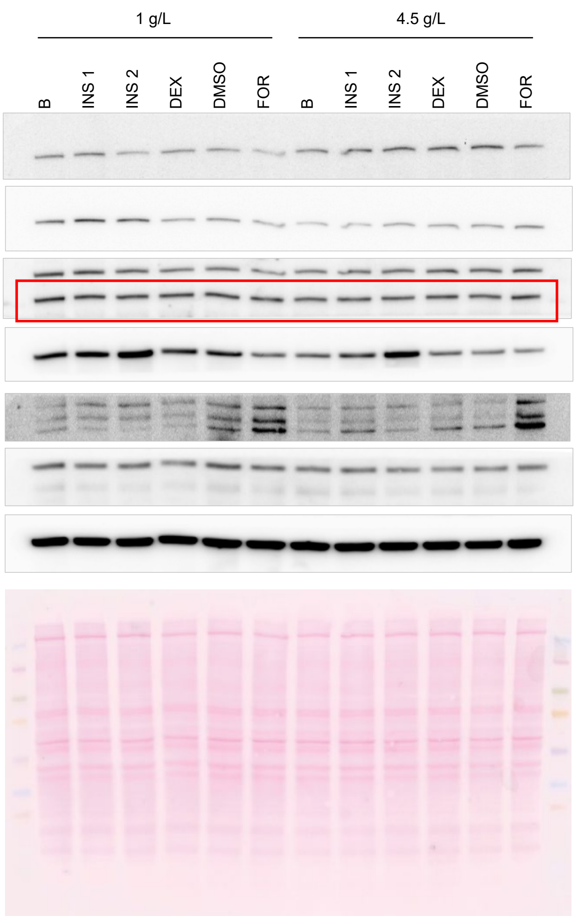

Donor 3 (IDF6)

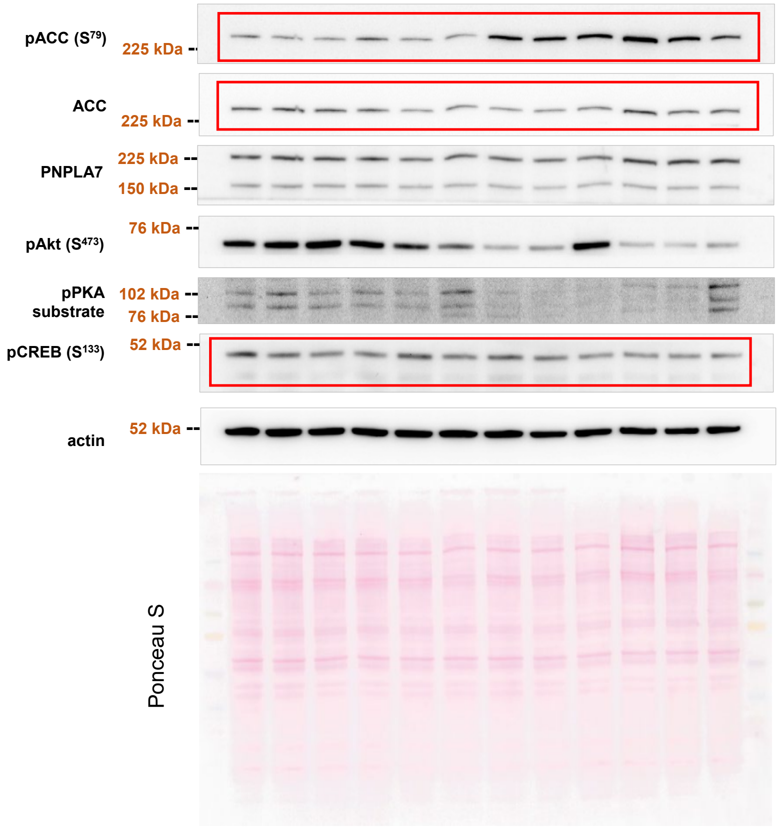

Donor 2 (IDF5)

The frame shows the blots that are presented in the figure.

Figure 4

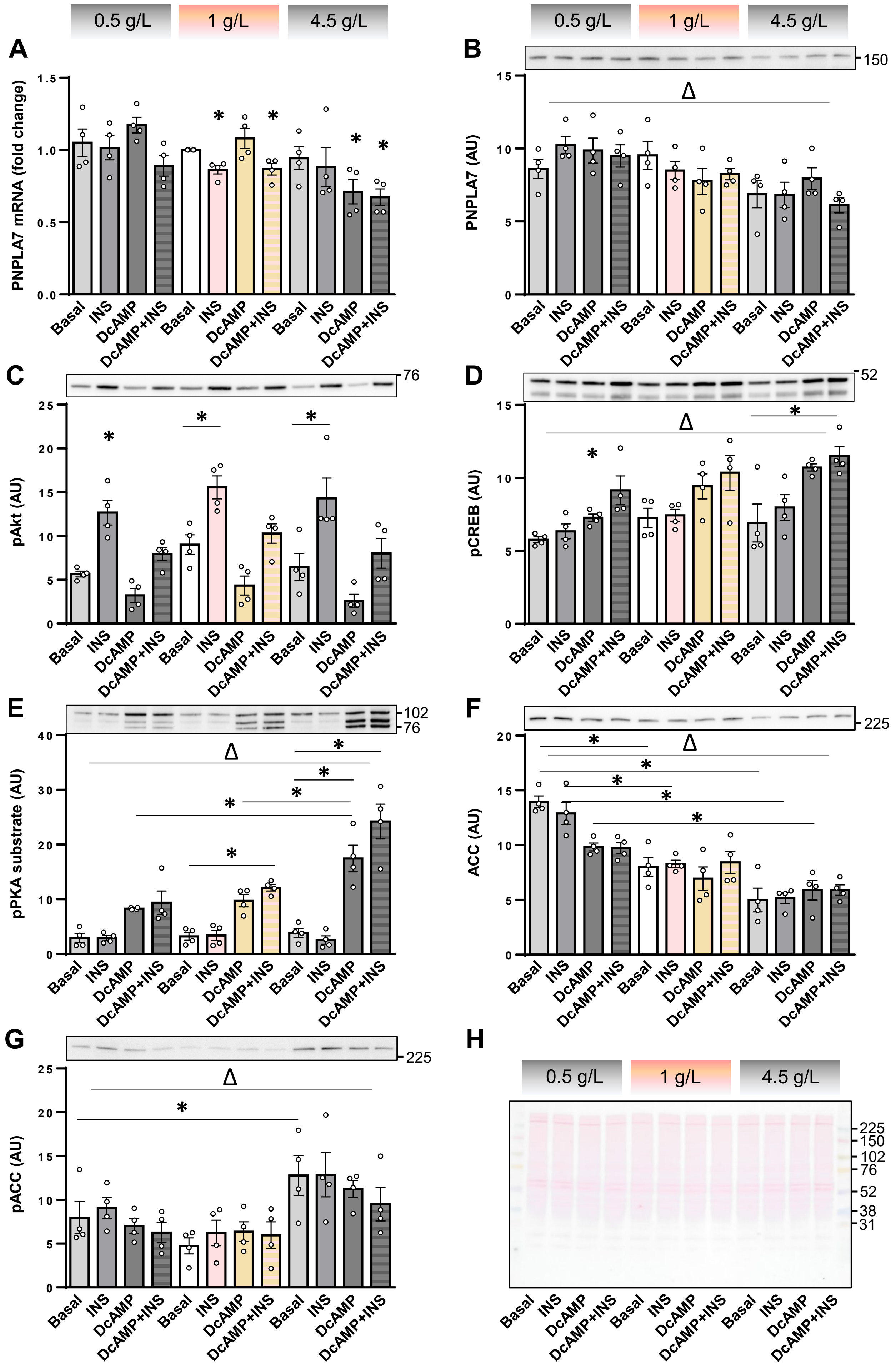
A
B
C
D
E
F
G
H
0.5 g/L
1 g/L
4.5 g/L
PNPLA7 mRNA (fold change)
PNPLA7 (AU)
pAkt (AU)
pCREB (AU)
pPKA substrate (AU)
ACC (AU)
pACC (AU)
Basal
INS
DcAMP
DcAMP+INS
150
76
52
102
225
38
31
Δ
*

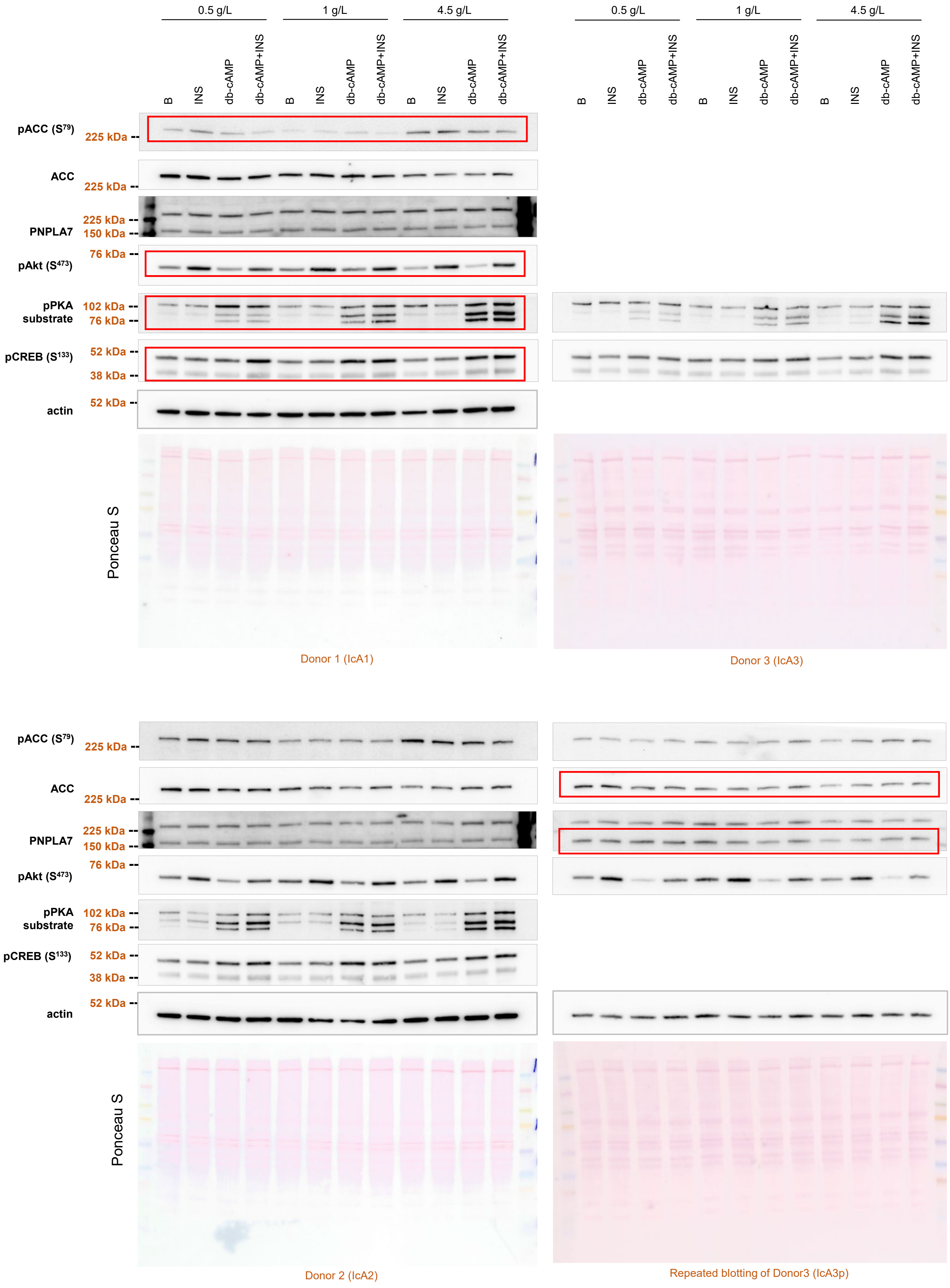

The frame shows the blots that are presented in the figure.

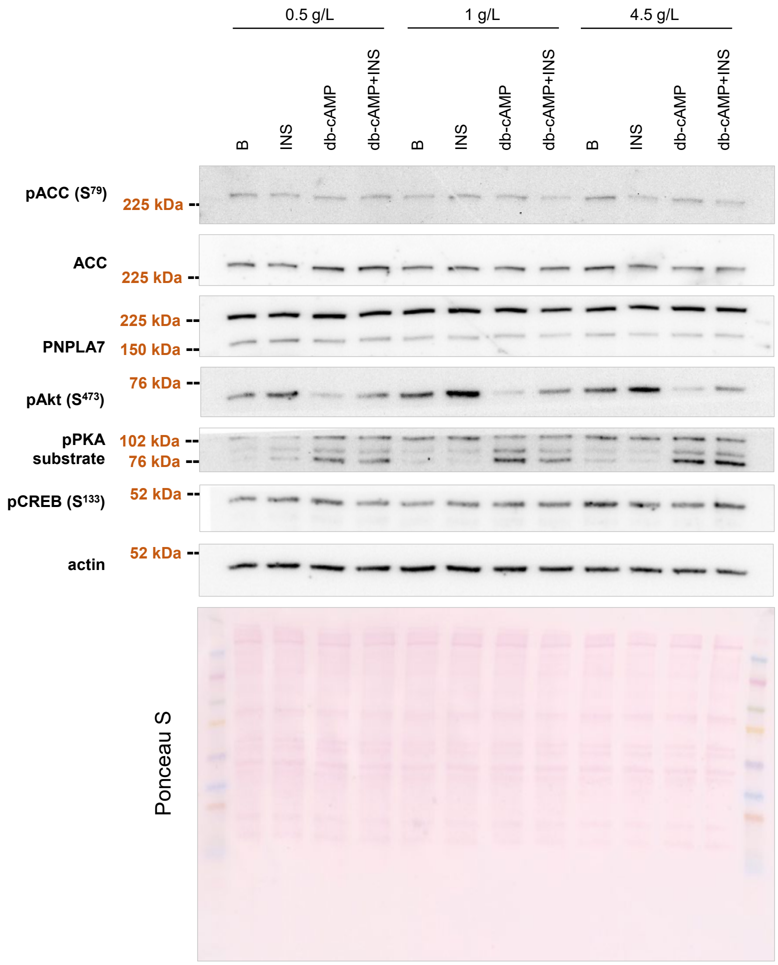

Sigma donor – series of parallel samples 1 (membrane IcA4)

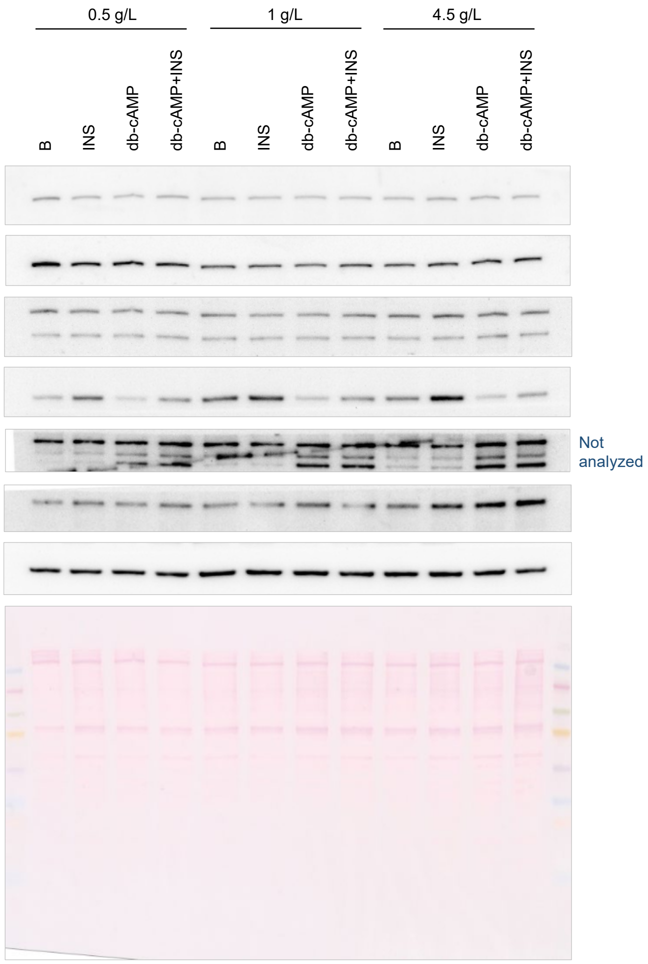

Sigma donor – series of parallel samples 3 (membrane IcA6)

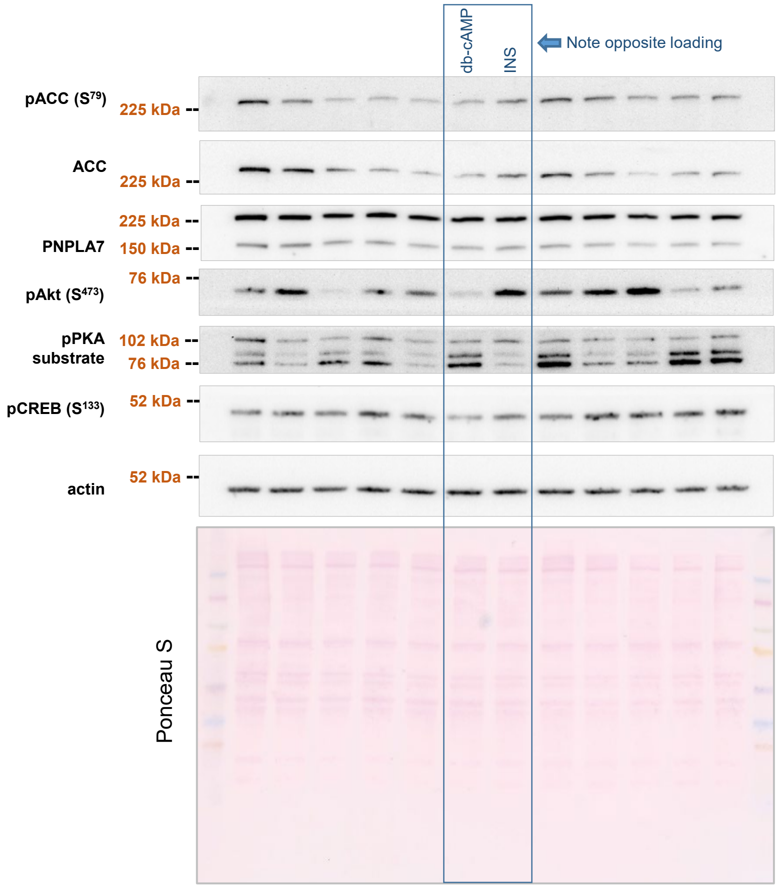

Sigma donor – series of parallel samples 2 (membrane IcA5)

For each treatment the average value of parallel samples from all three series was calculated and considered as the 4. donor in statistics (in addition to the donors 1-3 from the previous page).
